# Adherence and associated factors to iron and folic acid supplementation among pregnant women attending antenatal care in public hospitals of Dire Dawa, Eastern Ethiopia

**DOI:** 10.18332/ejm/138595

**Published:** 2021-08-25

**Authors:** Yonatan Solomon, Alekaw Sema, Tamiru Menberu

**Affiliations:** 1 Department of Nursing, College of Medicine and Health Sciences, Dire-Dawa University, Dire Dawa, Ethiopia; 2 Department of Midwifery, College of Medicine and Health Sciences, Dire-Dawa University, Dire Dawa, Ethiopia; 3 Department of Medical Laboratory, College of Medicine and Health Sciences, Dire-Dawa University, Dire Dawa, Ethiopia

**Keywords:** pregnant women, anemia, adherence, Ethiopia, Dire Dawa, iron and folic acid

## Abstract

**INTRODUCTION:**

Worldwide, 41.8% of pregnant women have anemia. Nationally, only 0.4% of pregnant women take the recommended 180-day iron supplement for more than 90 days. This study aimed to determine adherence to iron and folic acid supplements and factors affecting it among pregnant women attending antenatal care in public hospitals of Dire Dawa City, eastern Ethiopia.

**METHODS:**

An institutional-based cross-sectional study design was conducted from 1 January 2019 to 30 June 2019. In all, 416 pregnant women were selected using a systematic random sampling method. Data were collected using an interviewer-administered questionnaire and analyzed using SPSS version 22.00. Bivariate and multivariable logistic regression with a 95% confidence interval was done, and variables at a p<0.05 were considered statistically significant to the outcome variable.

**RESULTS:**

The study revealed that 71.8% of pregnant women have adhered to iron/folic acid supplements. Pregnant women who had ≥4 antenatal care visits (AOR=3.15; 95% CI: 1.16–9.05), got advice about iron/folic acid supplementation (AOR=3.12; 95% CI: 1.15–5.29), good knowledge about iron/folic acid supplementation (AOR=3.56; 95% CI: 1.42–8.54), good knowledge about anemia (AOR=5.22; 95% CI: 2.06–8.33), and currently anemic (AOR=2.58; 95% CI: 2.38–9.61) were significantly associated with adherence of iron/folic acid supplementation.

**CONCLUSIONS:**

The adherence of iron/folic acid supplementation of pregnant women was good. Getting advice about iron/folic acid supplementation, ≥4 antenatal care visits, having good knowledge about iron/folic acid supplementation and anemia, and currently anemic, were factors associated with adherence to iron/folic acid supplementation.

## INTRODUCTION

Physiological changes during pregnancy, fetal growth, and development, increase the need for iron and folic acid. The increased demand for these nutrients cannot be met by diet alone as the bioavailability of nutrients in pregnant women declines. Iron and folic acid deficiency can occur if food is not replaced with iron and folic acid tablets during pregnancy^1^.

Anemia during pregnancy is diagnosed as a haemoglobin level less than 11 g/dL for mild anemia, between 7–9.9 g/ dL for moderate anemia, and less than 7g/dL considered as severe anemia and usually due to iron deficiency^2^.

To reduce the risk of maternal iron deficiency anemia, the World Health Organization (WHO) recommends 60 mg of iron and 400 μg folic acid (IFA) supplements during pregnancy, starting with antenatal care as soon as possible^3,4^.

Worldwide, 1.62 billion people suffer from anemia, which is 24.8% of the world’s population. An estimated 56.4 million pregnant women suffer from anemia, of which 17.2 million are in African countries^5^. Daily iron and folic acid supplementation (IFAS) during pregnancy reduces the risk of all types of maternal anemia by 70% and iron deficiency anemia by 57% at term^3^. According to 19 African countries, the national demographic health survey dataset analysis shows that taking IFAS for 90 days during pregnancy can reduce the risk of neonatal mortality by 34%.

The 2016 Ethiopian demographic health survey (EDHS) found that only 5% of pregnant mothers consumed iron with a folic acid tablet for 90 days, and 58% of pregnant mothers did not take iron with a folic acid tablet during pregnancy^6^. According to the 2016 EDHS, 24% of women of reproductive age have anemia^6^. Anemia during pregnancy can severely affect both the mother and the fetus^7^. Overall, anemia accounts for 18% of perinatal deaths, 19% of premature births, and 12% of low birth weight in low- and middle-income countries^8^.

Numerous studies have reported that the use of any antenatal iron and folic acid supplementation during pregnancy reduces the risk of early neonatal and childhood mortality by preventing maternal anemia, low birth weight, and preterm delivery^9-11^.

IFA supplementation to pregnant women has been implemented at the facility and community level in every region of Ethiopia to achieve the WHO recommended level^12^. In contrast to such effort, daily iron supplementation coverage in Ethiopia is limited due to lack of compliance, the safety of the drug, and the volatile availability of drugs at the community level^13-15^.

Various studies have revealed that sociodemographic, maternal, and health service-related factors were affecting IFAS adherence. Among factors that are significantly associated with adherence to IFAS are: age^16-18^, educational level^18-21^, gravidity^18^, employment status^21^, residence^19,22,23^, monthly income^21^, knowledge of anemia and iron folate tablets^15-18,20,24,25^, number of ANC visits^15,19,22,25^, early ANC registration^17-19,22,23^, taking a number of tablets at each visit^22^, tablets taken when sick^21^, reports of side effects^21^, receiving information about the benefit of the tablets^17,25^, counseling on nutritional intake^15,16,23,24^, history of anemia^19,20,22^, and family support^23^ .

IFAS adherence level ranges from 3.5%^25^ to 76%^26^ in Ethiopia, which varies greatly in different geographical settings and different periods. The majority of previous studies were conducted in rural settings and community based on a small sample size, and the reports were also inconsistent.

As Dire Dawa is one of the two administrative cities found in Ethiopia next to Addis Ababa and as there is limited evidence from this study area, this study aimed to assess the magnitude of adherence to IFAS and its associated factors among pregnant women in attending ANC in Dire Dawa public hospitals, in eastern Ethiopia.

## METHODS

### Study area and period

The study was conducted in Dire Dawa city administration Dil Chora Referral Hospital (DCRH) and Sabiyan General Hospital (SGH) from 1 January 2019 to 30 June 2019. Dire Dawa city is one of the administrative cities of Ethiopia located 515 km from Addis Ababa, the capital city of Ethiopia. It has an estimated total population of about 0.5 million, of which 51.6% are females. The city administration has six hospitals (two governmental and four private hospitals) and eight health centers. Both hospitals offer health services including maternal and child health services. Some of these are inpatient, outpatient, antenatal care, delivery, postnatal care, and family planning services.

### Study design and population

An institution-based cross-sectional study was conducted to assess the magnitude of adherence and associated factors to iron and folic acid supplements among pregnant women attending antenatal care in Dire Dawa city public hospitals, eastern Ethiopia. Pregnant women who had at least one ANC visit in the hospitals and previously given 60 mg iron with 0.4 mg folic acid tablets for at least one month before the interview date were included while pregnant women with a mental disorder, unable to hear and/or speak, or very sick, were excluded from the study.

### Sample size determination and procedure

The sample size was determined by using the single population proportion formula:

$$n_{f}=\frac{{\left(Z_{\frac{a}{2}}\right)}^{2}p\left(1-p\right)}{d^{2}}$$

based on the following assumptions: $Z_{\frac{a}{2}}$ (95%) confidence interval, *p* (44%) magnitude of IFAS from the previous study conducted in Debre Tabor^27^, and *d* (5%) margin of error; the final sample size was 416.

In the study area, there are two governmental hospitals (SGH, and DCRH), and these two governmental hospitals were selected purposively. The study participants were allocated to the two hospitals proportionally based on the monthly total number of ANC attendants in the last year’s quarterly report of the same periods giving 268 for DCRH and 148 for SGH. A systematic sampling technique was then used to select the study participants from the ANC attendants. A sampling interval (k=2) was used for each hospital to select pregnant women, where the first pregnant woman was selected randomly. Finally, we interviewed the study participants at every two intervals among ANC service users.

### Data collection and quality control

The data collectors were five Diploma nurses. The data were collected using face-to-face interviews and chart review through a structured interviewer-administered questionnaire under the supervision of two Master’s graduates. The questionnaire was developed after reviewing the literature. First, the questionnaire was developed in English and translated to the local languages (Amharic, Oromiffa, and Aff-somalli) then translated back to English to keep its consistency. An intensive two days of training was given to the data collectors and supervisors. The questionnaire was pretested on 5% of the sample size (21) before the actual data collection period.

To ensure the quality of data, regular supervision, and checking of the filled in questionnaires for completeness and accuracy were performed on regular basis by the supervisors. Data were cleaned, and double data entry was done.

### Operational definitions

#### Adherence to IFAS

Mothers were said to adhere to IFAS if they took the supplement at least 4 days a week during the 1 month preceding the study^28^.

#### Good knowledge about IFAS

Those who scored greater than or equal to the mean value of correct responses from 7 item questions prepared to assess comprehensive knowledge of IFAS of the respondents were deemed to have good knowledge about IFAS.

#### Good knowledge about anemia

Those who scored greater than or equal to the mean value of correct responses from 9 questions prepared to assess comprehensive knowledge of anemia of the respondents were deemed to have good knowledge about anemia.

### Ethical issues

The study strictly followed the principles outlined in the Declaration of Helsinki in addition to obtaining ethical clearance from Dire-Dawa university Department of Nursing on the date of 12/12/2018 with Ref No: DN/22/18. All participants were informed why the research is being conducted and anonymity was assured, and how the data collected was going to be stored. Consent was obtained from the study participants before study commencement. The data collectors also discussed the issue of privacy, the confidentiality of the information obtained during the interview, and both verbal and written informed consent was obtained from respondents. Respondents were provided with an information sheet which contained the following main points: purpose/aim of the study, procedure and duration of the interview, risks and benefits of participation, confidentiality and rights of the participants, and contact address of the researcher for any questions, and finally declaration of informed voluntary consent.

### Statistical analysis

After the data were checked for completeness, they were coded and entered to EpiData (Classic) Entry version 3.1 and then exported to SPSS version 21 for analysis. The presentation of the data was done by using frequency distribution, percentage, and mean. Data cleaning and assumption checking was performed before proceeding for analysis. Binary and multiple logistic regression analyses were done to determine whether the independent variables predict the dependent variable. Variables with a p<0.2 during a bivariable analysis were incorporated into the multivariable logistic regression to control for the possible effects of confounders. The adjusted odds ratio (AOR) with 95% confidence interval (CI) was computed to see the strength of the association and a p<0.05 was considered statistically significant.

## RESULTS

### Sociodemographic characteristics of study participants

A total of 401 study participants were involved with a response rate of 96%. The age of the respondents was 15– 39 years with a mean of 27.2 (±5.5) years. A large proportion (43.3%) of the study participants were within the age range 25–29 years. The majority (72.6%) of the respondents were married with a family size of 4–6 (57.4%) persons. Regarding educational level, 149 (37.3%) participants attended secondary level education. A total of 174 (43.3%) of the respondents were governmental employees (Table 1).

**Table 1:** Distribution of sociodemographic characteristics of pregnant women attending ANC in Dire Dawa Public Health Hospitals, Eastern Ethiopia, 2019 (N=401)

*Characteristics*	*n*	*%*
**Age** (years) 15–19	7	1.74
20–24	113	28.2
25–29	174	43.3
30–34	95	23.7
≥35	12	3.06
**Marital status**		
Married	291	72.6
Single	63	15.6
Divorced	39	9.7
Widowed	8	2.1
**Education level**		
Unable to read and write	13	3.2
Able to read and write	67	16.7
Primary education	85	21.2
Secondary education	149	37.3
College and above	87	21.6
**Family size** (persons)		
1–3	106	26.3
4–6	230	57.4
>6	65	16.3
**Occupation**		
Housewife	137	34.1
Daily laborer	37	9.4
Government employee	174	43.3
Merchant	53	13.2

### Obstetric and health-related characteristics of study participants

Two hundred and thirty-four (58.4%) of the respondents were multigravidas whereas 193 (48.1%) were nulliparas. A total of 287 (71.6%) of the respondents were in their third trimester of the pregnancy during the data collection period while 178 (44.5%) of participants start their ANC follow-up from the first trimester. The majority (84.6%) of pregnant women were attending four or more ANC visits.

The majority of the study participants (71.6%) had been advised about the use of IFAS. Most pregnant women (78.5%) had no history of anemia in their lifetime but 103 (25.8%) of pregnant women had anemia currently. More than half (69.7%) of the study participants had good knowledge about anemia and 67.2% good knowledge about IFAS (Table 2).

**Table 2:** Obstetric and health-related characteristics of pregnant women attending ANC service attending Antenatal Clinic in Dire Dawa Public Health Hospitals, Eastern Ethiopia, 2019 (N=401)

*Characteristics*	*n*	*%*
**Gravidity**		
Primigravida	167	41.6
Multigravida	234	58.4
**Parity**		
Nullipara	193	48.1
Primipara	89	22.1
Multipara	119	29.8
**Alive child**		
No	164	41.0
Yes	237	59.0
**Number of ANC visits**		
2–3	62	15.4
≥4	339	84.6
**Time of first ANC**		
First trimester	178	44.5
Second trimester	126	31.4
Third trimester	97	24.1
**Trimester for current ANC**		
Second	114	28.4
Third	287	71.6
**Advised about IFAS**		
No	115	28.7
Yes	286	71.3
**History of anemia**		
No	315	78.5
Yes	86	21.5
**Anemia currently**		
No	298	74.2
Yes	103	25.8
**Good knowledge of anemia**		
No	122	30.3
Yes	279	69.7
**Good knowledge of IFAS**		
No	132	32.8
Yes	269	67.2

### Magnitude of adherence to IFAS

The current study revealed that the majority (71.8%) of pregnant women adhere to IFAS.

### Associated factors of adherence to IFAS

Multivariable logistic regression analyses were conducted to identify factors associated with IFAS and according to the results, knowledge about anemia and IFAS, number of ANC visits, current anemia status, and advised about IFAS were found to be significantly associated with adherence to IFAS.

Pregnant women who had ≥4 ANC visits were three times more likely to adhere to IFAS compared to those who had 2–3 ANC visits (AOR=3.15; 95% CI: 1.16–9.05). Mothers who got advice about IFAS were 3 times more likely to be adherent to IFAS than those who did not get advice about IFAS (AOR=3.12; 95% CI: 1.15–5.29).

Mothers who had good knowledge about IFAS were 3 times more likely to adhere to IFAS compared to their counterparts (AOR=3.56; 95% CI: 1.42–8.54). Mothers who had good knowledge about anemia were 5 times more likely to adhere to IFAS than those who had poor knowledge (AOR=5.22; 95% CI: 2.06–8.33), and those mothers who were currently anemic were 2 times more likely to adhere to IFAS than those who were not currently anemic (AOR=2.58; 95% CI: 2.38–9.61) (Table 3).

**Table 3:** Factors associated with adherence to iron and folic acid supplementation among pregnant women attending antenatal care clinics in Dire Dawa Public Health Hospitals, Eastern Ethiopia, 2019 (N=401)

*Characteristics*	*Adherence*		*OR (95% CI)*	*AOR (95% CI)*	*p*
	*Yes n (%)*	*No n (%)*			
**Number of children**					
1–3	54 (51.2)	52 (48.8)	2.13 (1.41–7.25)	1.2 (0.43–2.42)	0.524
4–6	102 (44.4)	128 (55.6)	3.21 (0.24–2.12)	1.26 (0.13–3.02)	0.422
≥7	27 (41.2)	38 (58.8)	1	1	
**Knowledge about anemia**					
Good	208 (74.7)	71 (25.3)	3.41 (1.34–7.41)	5.2 (2.06–8.33)	0.002
Poor	33 (27.4)	89 (72.6)	1	1	
**Knowledge of IFAS**					
Good	186 (69.3)	83 (30.7)	4.42 (1.26–5.13)	3.56 (1.42–8.54)	0.002
Poor	37 (28.3)	95 (71.7)	1	1	
**Number of ANC visits**					
≥4	212 (62.4)	127 (37.6)	5.48 (3.44–6.26)	3.15 (1.16–9.05)	**0.001**
2–3	19 (31.3)	43 (68.7)	1	1	
**Current anemia**					
No	77 (25.7)	221 (74.3)	1	1	
Yes	67 (64.7)	36 (35.3)	1.08 (2.11–3.37)	2.58 (2.38–9.61)	0.001
**Advised about IFAS**					
Not advised	40 (35.2)	75 (64.8)	1	1	
Advised	196 (68.4)	90 (31.6)	1.97 (1.01–3.15)	3.12 (1.15–5.29)	0.003

AOR: adjusted odds ratio.

## DISCUSSION

The results of this study revealed that 71.8% (95% CI: 30.4–40.7) of pregnant women were adherent to IFAS. This finding is consistent with a study conducted in Mizan Aman town (70.6%), Eritrean refugee camp (64.7%), and Akaki Kality Addis Ababa (60%)^16,21,29^. However, this finding was higher than other studies conducted in different areas of Ethiopia; such as in Assela town (59.8%), Debre Markos town (55.5%), Gondar, northwest Ethiopia (55%), and Aykel town (47.6%)^18,22,28,30^. The possible reasons might be due to differences in the study setting and time, as our study was hospital-based and recent whereas these studies were community-based, urban, had accessibility to health services and awareness of IFAS, and higher literacy level for IFAS than the current study population (67.2%).

According to the current study findings, the number of ANC visits had a significant association with adherence to IFAS. Pregnant women who had ≥4 ANC visits were three times more likely to adhere to IFAS than those who had 2–3 ANC visits. This finding is consistent with the study done in the western zone of Tigray, Assela town, Debre Tabor general hospital, and Mizan Aman town^18,19,27,29^. The possible explanation could be that pregnant women who had more ANC visits acquired better knowledge of the perceived risks and the benefits of IFAS to prevent anemia during pregnancy.

The current study indicated that advice about IFAS had a significant association with IFAS adherence status. Pregnant women who got advice about IFAS were three times more likely to be adherent to IFAS than those who did not. This finding was in line with a study done at Uganda, Misha district, Mizan Aman, and Debre Tabor general hospital^15,27,29,31^. Getting advice at the time of ANC may increase the level of knowledge, positive attitude, and practice towards IFAS adherence.

Also, the current study indicated that having good knowledge about IFAS had a significant association with adherence to IFAS. Pregnant women who had good knowledge about IFAS were three times more likely to be adherent to IFAS than those who had poor knowledge. This finding is similar to other studies conducted in Debre Tabor general hospital, Mecha district, Misha district, and Goba district^15,17,20,27^. Knowledge may increase the level of awareness about IFAS, and, in turn, it will increase the positive attitude and practice towards adherence to IFAS.

Another factor that had a significant association with IFAS in the present study was knowledge about anemia. Pregnant women who were knowledgeable about anemia were five times more likely to be adherent to IFAS than those who were not knowledgeable about anemia. This finding is consistent with other studies done in southeast Ethiopia, western Iran, and Aykel town^17,30,32^.

The last variable to be significantly associated with adherence to IFAS was current anemia. Pregnant women who had anemia currently were two times more likely to be adherent to IFAS than those pregnant women who did not. It is consistent with the study conducted in Debre Tabor general hospital, the northwestern zone of Tigray, India, and Mecha district^19,20,27,33^. The perceived risk of complications of anemia may be high in pregnant women who have current anemia.

### Limitations

Since the study was based on the previous one-month intake of IFA tablets, it might be subject to potential recall bias. Another limitation of the study might be that IFA adherence was determined by the pregnant women’s response (self-reported adherence measuring method) which might not reflect the actual adherence rate of the source population. Also, the estimation of IFAS adherence by the self-report method may underestimate the prevalence of non-adherence when compared with objective measures like pill counts or biological assays medication adherence measures.

## CONCLUSIONS

The adherence of IFAS among pregnant women in the study area was high. The number of ANC visits, advice about IFAS, knowledge about IFAS, and anemia, and current anemia status were independent predictors of adherence to IFAS.

## Data Availability

The data supporting this research are available from the authors on reasonable request.
